# Supplementary material for: Characterization of Lung and Oral Microbiomes in Lung Cancer Patients Using Culturomics and 16S rRNA Gene Sequencing
Source: Microbiol Spectr. 2023 Apr 24;11(3):e00314-23. doi: 10.1128/spectrum.00314-23 (PMC10269771; doi:10.1128/spectrum.00314-23)
Supplement: Supplemental file 1 — Fig. S1 to S4 and Tables S1 and S2. Download spectrum.00314-23-s0001.pdf, PDF file, 0.6 MB [file spectrum.00314-23-s0001.pdf]

Characterization of lung and oral microbiome in lung cancer patients by cultruomics and 16S rDNA sequencing

Yifan Sun[1,#], Yuejiao Liu[1,#,] Jianjie Li[2], Yafang Tan[1], Tongtong An[2], Minglei Zhuo[2], Zhiyuan Pan[1], Menglei Ma[2], Bo Jia[2], Hongwei Zhang[2], Ziping Wang[2,*], Ruifu Yang[1,*], Yujing Bi[1,*]

1. State Key Laboratory of Pathogen and Biosecurity, Beijing Institute of Microbiology and Epidemiology, Beijing 100071, China;
2. Department of thoracic oncology, Peking University Cancer Hospital, Beijing 100142, China

#These authors contributed equally to this work.
* Corresponding authors: Ziping Wang (wangzp2007@126.com)
Ruifu Yang (13801034560@163.com)
Yujing Bi (byj7801@sina.com);

Fig S1. Bacteria identified from the the oral samples. **A)** Phylogenetic tree and **B)** proportion of 156 bacterial species isolated from the oral samples listed according to their phylum.

A

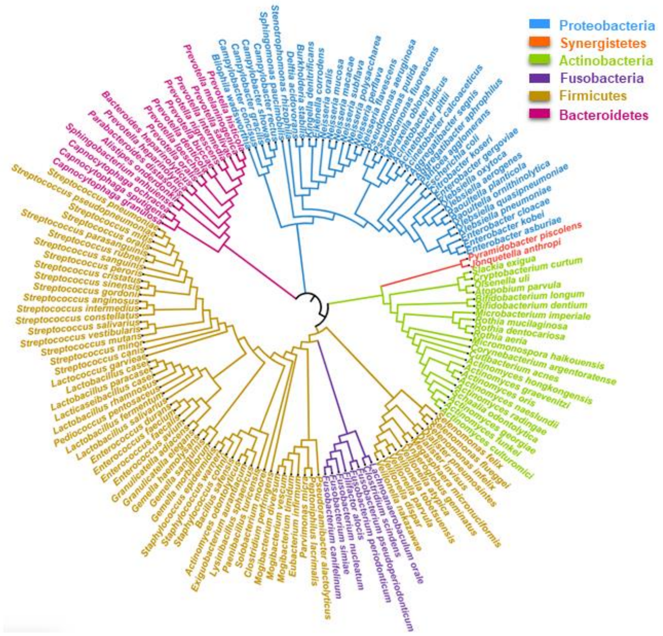

B

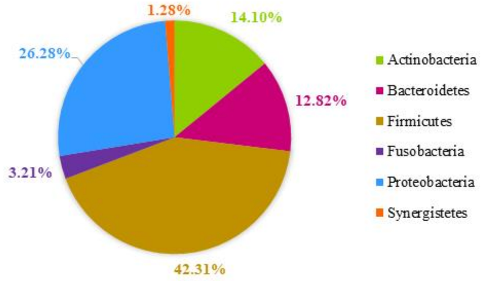

Fig S2. Differentially abundant taxonomy between lung and oral group. **A** Taxonomic composition at genera level in C, H, and O groups. **B** Notched box plots illustrating the differences of the significantly other 6 genus relative abundances in three different anatomy sites. *$p < 0.05$

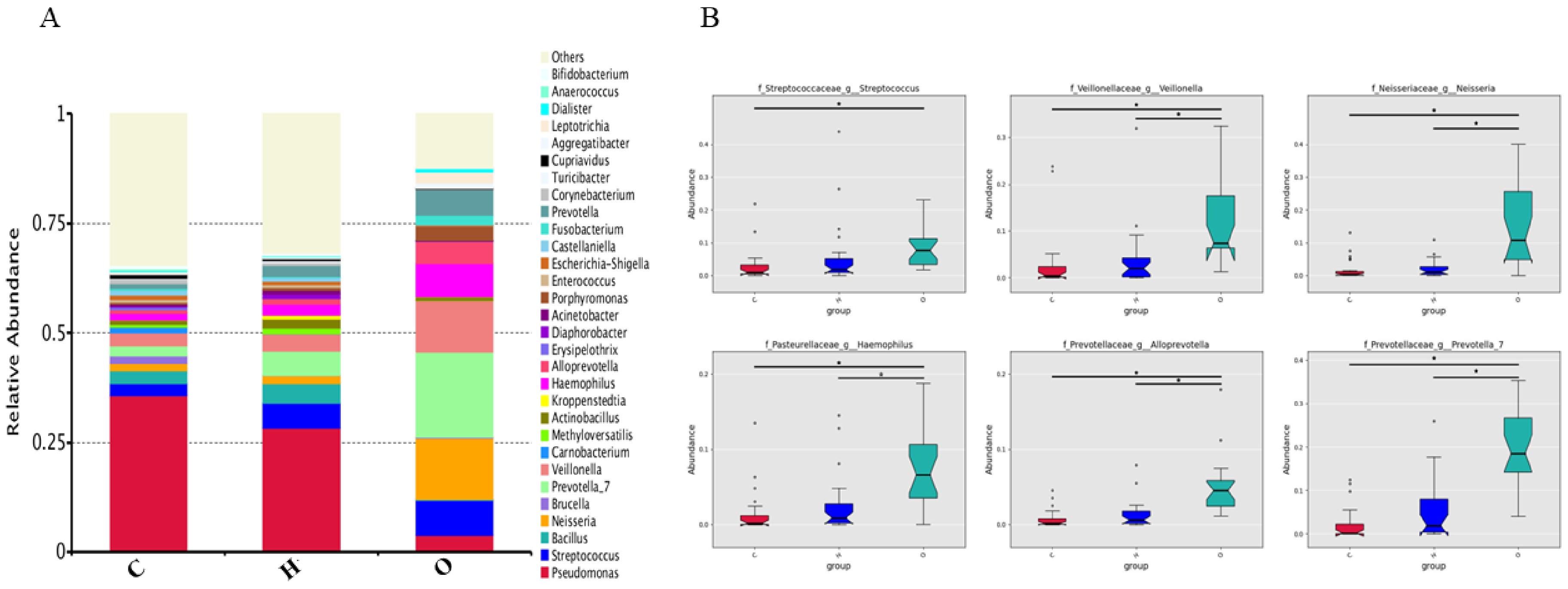

Fig S3 Comparison of the alpha and beta diversity for microbiota from cancer and healthy lungs. **A** Comparison of Shannon and Chao1 index between C and H group is shown. **B** PCoA analysis of C and H in BALF samples. ANOSIM was performed to test statistically whether there is a significant difference.

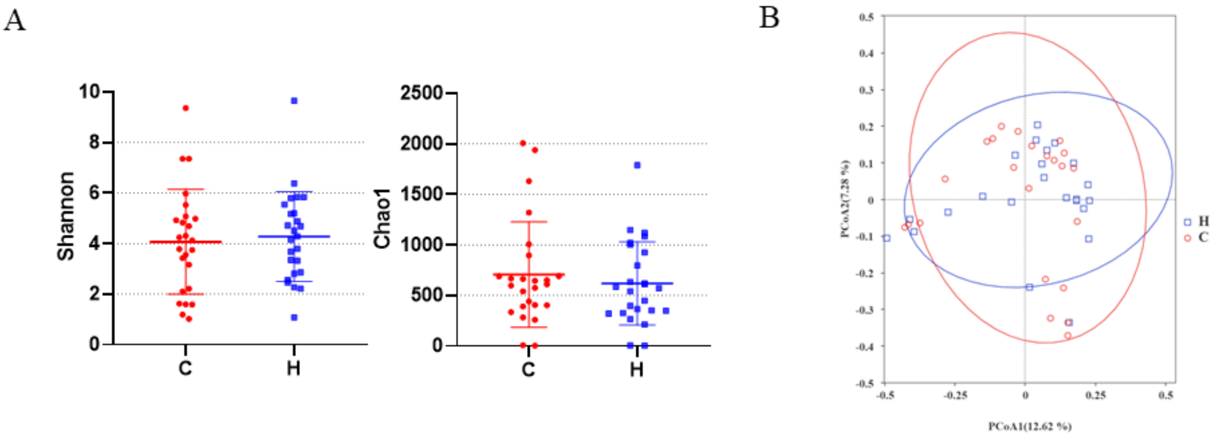

Fig S4. Characterization of the lung microbiota in the NSCLC and SCLC and differences in adenocarcinoma and squamous cell carcinoma. **A** Comparison of Shannon and Chao1 index between NSCLC and SCLC group is shown. **B** PCoA analysis of NSCLC and SCLC controls in lung samples. ANOSIM was performed to test statistically whether there is a significant difference. **C** Comparison of Shannon and Chao1 index between ADC and SCC group is shown. **D** PCoA analysis of ADC and SCC controls in lung samples. ANOSIM was performed to test statistically whether there is a significant difference.

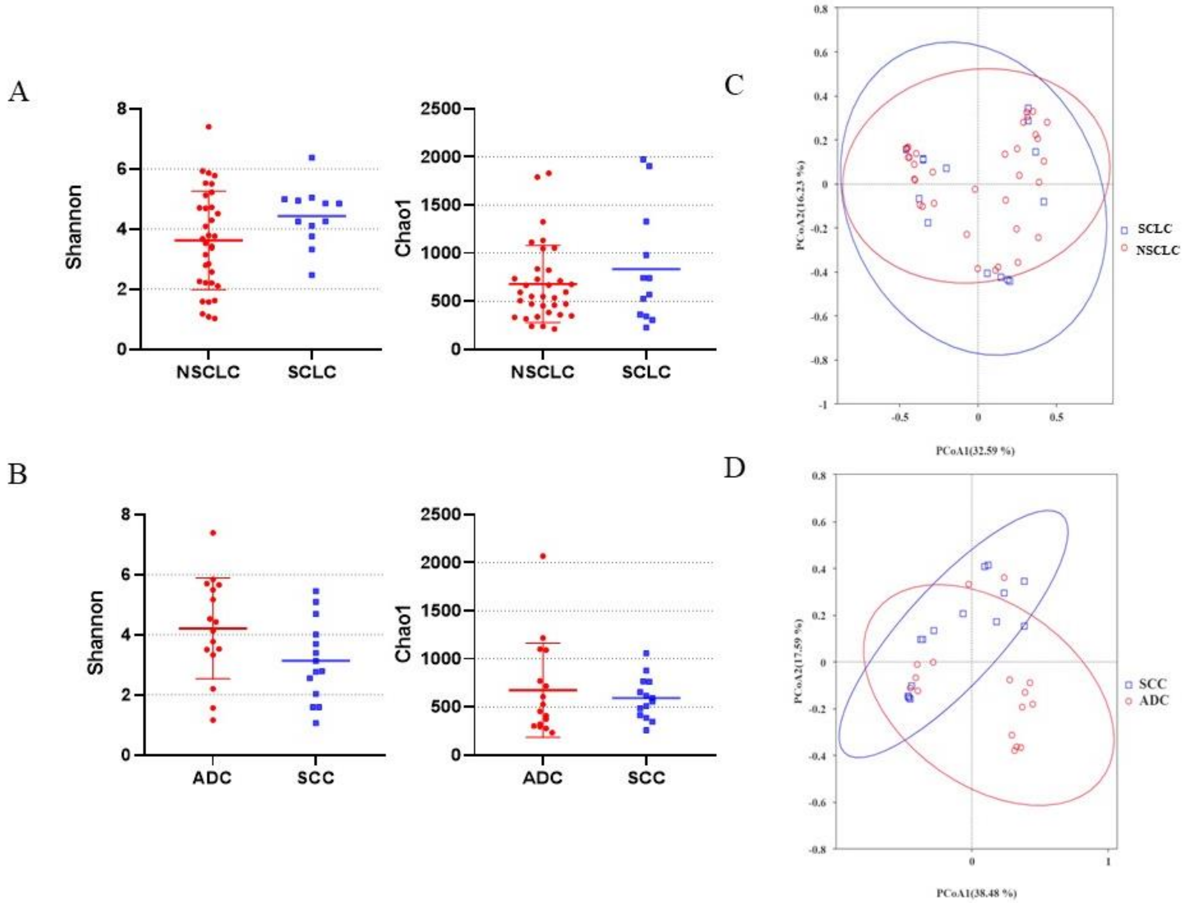

Table S1. Characteristics of the fifteen novel bacterial species.

Name	Source	Group	Identity	Closest relatives	Phylum
new.1	9	O	97.74	*Parvimonas parva*	Firmicutes
new.2	10	C	97.99	*Parvimonas micra*	Firmicutes
new.3	10	C	98.31	*Selenomonas timonae*	Firmicutes
new.4	10	C	91.94	*Denitrobacterium detoxificans*	Actinobacteria
new.5	12	C	98.03	*Parvimonas micra*	Firmicutes
new.6	12	O	96.08	*Petrimonas sulfuripCila*	Bacteroidetes
new.7	12	H	93.45	*Aminipila luticellarii*	Firmicutes
new.8	12	O	91.96	*Baileyella intestinalis*	Firmicutes
new.9	13	H	98.51	*Oribacterium asaccCarolyticum*	Firmicutes
new.10	13	O	91.98	*Denitrobacterium detoxificans*	Actinobacteria
new.11	15	C	97.43	*Selenomonas flueggei*	Firmicutes
new.12	17	C	96.50	*Pseudoramibacter alactolyticus*	Firmicutes
new.13	19	H	91.18	*Denitrobacterium detoxificans*	Actinobacteria
new.14	19	O	91.62	*Baileyella intestinalis*	Firmicutes
new.15	22	H	96.79	*Mobiluncus curtisii*	Actinobacteria

Abbreviations: C, H, O: samples from the cancerous site and contralateral healthy controls from lungs and the oral site, respectively, of patients with lung cancer.

Table S2. Demographics and clinical characteristics between NSCLC and SCLC.

Variable	NSCLC	SCLC	*p*-value
**N**	16	7	
**Age-mean (SD)**	65 (7.0)	67 (12.8)	0.605[a]
**pGender**			
Male, n (%)	9 (56%)	5 (72%)	0.824[b]
Female, n (%)	7 (44%)	2 (14%)	
**Smoking**			
Current or former Smoker, n (%)	10 (63%)	6 (86%)	0.535[b]
Never smoker, n (%)	6 (37%)	1 (14%)	
**Distant**			
MO	4 (25%)	2 (29%)	0.858[b]
M1	12 (75%)	5 (71%)	

[a]: *p* values were calculated by independent t test; [b]: *p* values were calculated by chi-square test.
